# Draft genomes and assemblies of the ectomycorrhizal basidiomycetes *Scleroderma citrinum* hr and *S. yunnanense* jo associated with chestnut trees

**DOI:** 10.7150/jgen.103481

**Published:** 2025-01-01

**Authors:** Haolin Zhang, Jiayi Han, Richard D. Hayes, Kurt LaButti, Igor Shabalov, Anna Lipzen, Kerrie Barry, Igor V. Grigoriev, Qing Zhang, Qingqin Cao, Huchen Li, Francis M. Martin

**Affiliations:** 1 Beijing Key Laboratory of New Techniques in Agricultural Application, Beijing University of Agriculture, Beijing 102206, China.; 2 U.S. Department of Energy Joint Genome Institute, Lawrence Berkeley National Laboratory, Berkeley, CA 94720, USA.; 3 Department of Plant and Microbial Biology, University of California Berkeley, Berkeley, CA 94720, USA.; 4 Université de Lorraine, INRAE, UMR 1136 Interactions Arbres/Microorganismes, 54280, Champenoux, France.; 5 Northwest Institute of Eco-Environment and Resources, Lanzhou, China.

**Keywords:** Boletales, ectomycorrhizal symbiosis, genome, *Scleroderma*, Sclerodermataceae.

## Abstract

The earthball *Scleroderma*, an ectomycorrhizal basidiomycete belonging to the Sclerodermataceae family, serves as a significant mutualistic tree symbiont globally. Originally, two genetically sequenced strains of this genus were obtained from fruiting bodies collected under chestnut trees (*Castanea mollissima*). These strains were utilized to establish *in vitro* ectomycorrhizal roots of chestnut seedlings. The genome sequences of these strains share characteristics with those of other ectomycorrhizal species in Boletales order, including a restricted set of genes encoding carbohydrate-active enzymes. The genome sequences presented here will aid in further exploring the factors contributing to the establishment of ectomycorrhizal symbiosis in chestnut trees.

*Scleroderma*, commonly known as earthballs, is a widely distributed ectomycorrhizal gasteromycetes genus that produces large, noticeable sporocarps or earthballs in various forest environments and areas adjacent to forests 1. The ectomycorrhizal status of* S. citrinum* and *S. yunnanense* has been confirmed and symbiosis can be established in* in vitro* experimental systems 2. *Scleroderma citrinum* belongs to the family Sclerodermataceae. This mushroom appeared early in the fruiting succession of ectomycorrhizal fungi. It is the primary colonizer of mining waste, enabling it to spread rapidly and colonize young root systems of numerous tree species. The genus *Scleroderma* is found worldwide, and* S. citrinum* is commonly recorded in temperate regions of the Northern Hemisphere (Fig. 1A) 3. Several host genera have been reported for *Scleroderma*, including poplars and eucalypts 4,5. Multiple studies have investigated the symbiotic relationships between *Scleroderma* species and their host plants, focusing on the growth and nutritional benefits of this relationship 3,6-8. Recently, the *S. citrinum* mycorrhizosphere has been studied as a model system to examine the impact of ectomycorrhizal symbiosis on the taxonomic and functional diversity of bacterial communities involved in mineral weathering 3,9,10.

In October 2018, *S. citrinum* hr. and *S. yunnanense* jo. strains were isolated from fruiting bodies growing under chestnut trees (*Castanea mollissima*) in Huairou and Jianou, China, respectively. These strains were cultured on agar plates containing the P20 medium at 25°C, and subsequent mycelial (monosporal) cultures were used to remove contaminants until identical mycelia were obtained. The identity of the purified strains was confirmed by sequencing the rDNA ITS region using the primers ITS4 and ITS5^11^. After molecular characterization, both strains were deposited at Beijing Key Laboratory of New Techniques in Agricultural Application with accession numbers 0322 for *S. citrinum* hr and 0323 for *S. yunnanense* jo. The growth of both strains on P20, MMN, and PDA media was compared, revealing that P20 medium was the most optimized for both strains (Fig. 1B).

Fresh mycelia were obtained from fungal colonies grown on the P20 medium and were snap-frozen in liquid nitrogen. The samples were subsequently ground using a TissueLyser LT (QIAGEN). DNA was isolated following the CTAB extraction protocol 12, and total RNA was isolated using an RNAeasy extraction kit (QIAGEN), according to the manufacturer's instructions. During the extraction procedures, RNase A or DNase I (both from Thermo Fisher) were used to purify DNA or RNA, respectively. Approximately 200 μg of DNA and 10 μg of total RNA were extracted from each strain.

The *S. citrinum* hr and *S. yunnanense* jo v1.0 genomes were sequenced from 100 μg of genomic DNA using the Pacific Biosciences sequencing platform (>10kb PacBio libraries with Blue Pippin size selection), assembled with Falcon v. 0.0.8^13^, polished with Arrow version SMRTLINK v8.0.0.80529 (https://www.pacb.com/support/software-downloads), and annotated using the MycoCosm developed by JGI^14^, information of genomic portals can be found in https://mycocosm.jgi.doe.gov/Sclcihr1 and https://mycocosm.jgi.doe.gov/Sclyun1. The Whole Genome Shotgun project has been deposited in GenBank under BioProjects PRJNA711063 and PRJNA1080752. To support gene prediction, mRNA sequences were obtained using Illumina RNA-Seq data assembled using Trinity v2.11.0^15^.

The assembly size of the Chinese strain hr of *S. citrinum* (77,47 Mbp), was higher than that of the previously sequenced European strain *S. citrinum* Foug A (i.e., 56,14 Mbp), whereas the number of genes was lower (10323 genes versus. 21012 genes) 16. We identified polymorphic content within the assembly and annotation of strain hr, which reflected significant separation of alleles. Many scaffolds were observed to be highly similar to larger scaffolds and were predicted to constitute alternate or secondary haplotypes. The assembly size of the* S. yunnanense* genome (45,55 Mbp) was lower than that of *S. citrinum* genomes, despite the fact that the number of genes was very similar between the two Chinese strains, specifically 10,323 versus. 10,194.

A restricted set of genes encoding carbohydrate-active enzymes (CAZymes) is a major trait in the evolution of ectomycorrhizal fungi from saprotrophic ancestors 16. The genomes of *S. citrinum* hr and *S. yunnanense* contain 244 CAZyme genes, whereas *the S. citrinum* FougA genome encodes 257 CAZyme genes. This repertoire of CAZyme genes acting on lignocellulose is much lower than that of their saprotrophic relatives in Boletales, such as *Coniophora* and *Serpula* species, as well as ectomycorrhizal fungi, such as *Suillus* and *Boletus* spp. (Fig. 2). In contrast, their CAZyme repertoire is very similar to that of *Pisolithus* species, which are also found in sandy soils with low soil organic matter content.

The current set of genome sequences will enable additional research into the molecular factors that drive the formation of ectomycorrhizal symbiosis in chestnut trees.

## Figures and Tables

**Figure 1:**
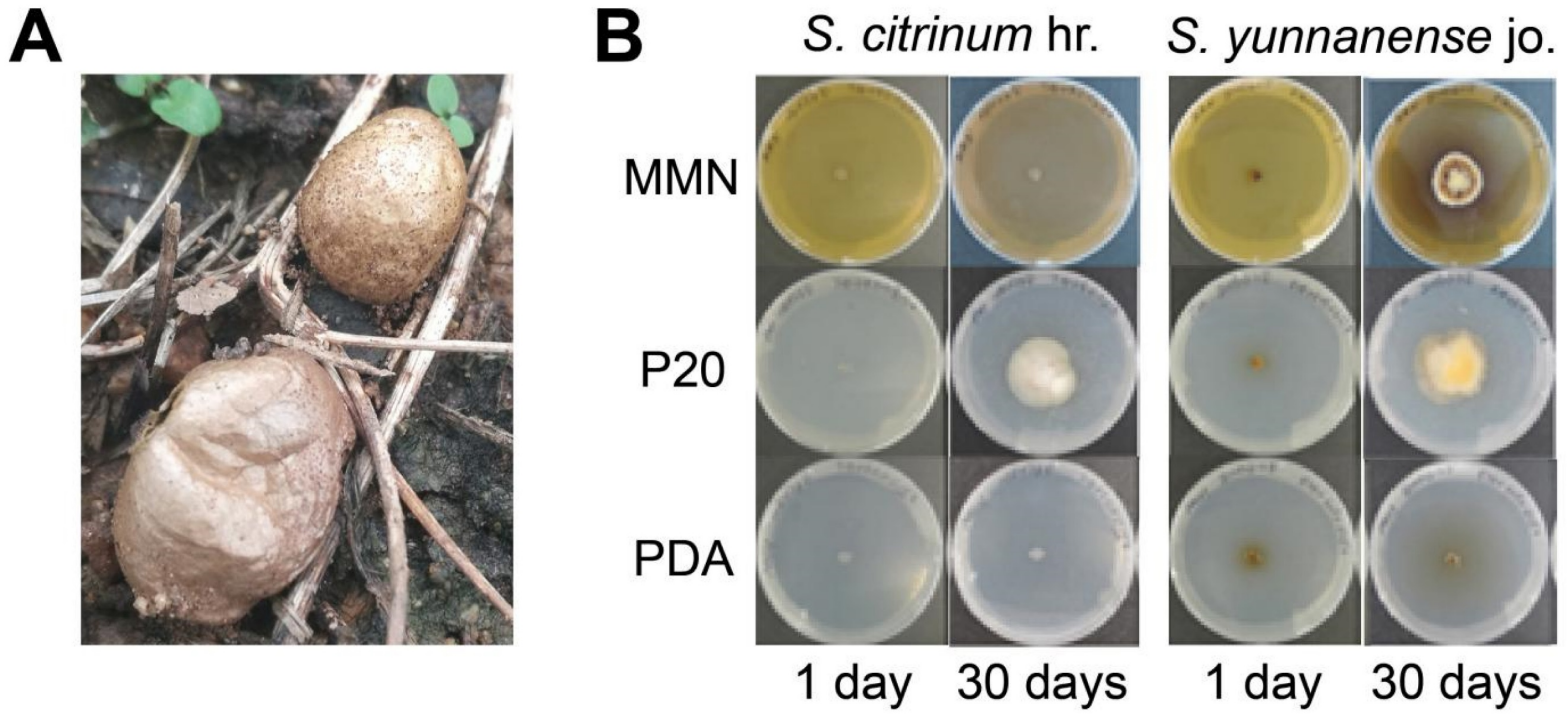
Basidiocarps (**A**) and vegetative mycelial cultures (**B**) of *S. citrinum* and *S. yunnanense.* A, natural habitate of *S. citrinum* hr. (B) Growth of *S. citrinum* hr and *S. yunnanense* jo strains on MMN, P20, and PDA solid media 1 or 30 days after inoculation.

**Figure 2:**
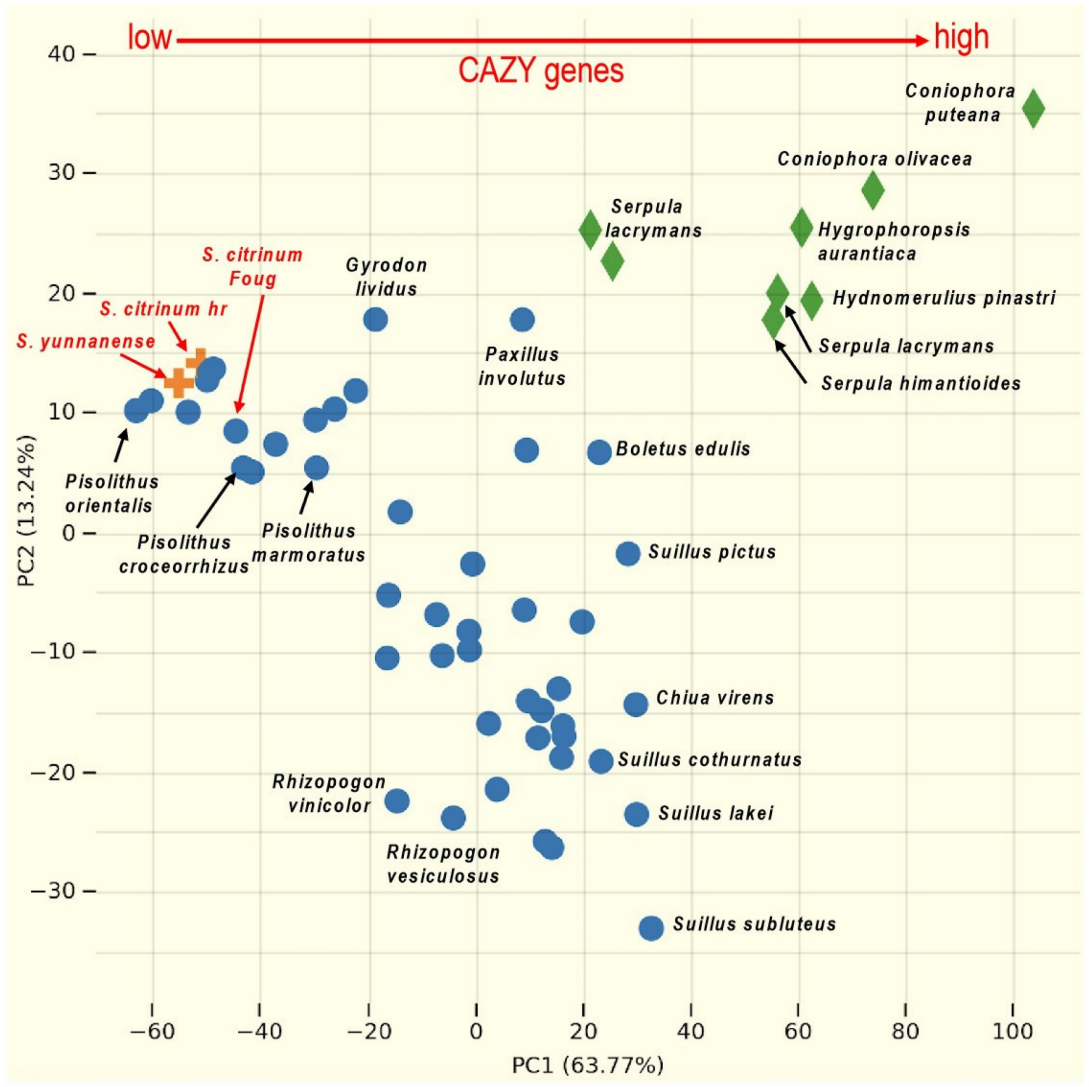
Differential distribution of CAZyme gene copy numbers in the genomes of 92 species of saprotrophic and ectomycorrhizal fungi in Boletales. Principal coordinate analysis of the total CAZyme gene copy numbers identified in saprotrophic and ectomycorrhizal fungi. Each symbol corresponds to the genome of the Boletales species available in the JGI MycoCosm database (mycocosm.jgi.doe.gov). The lifestyle of the sequenced species is indicated by different colored species names (green for saprotrophic species and blue and red for ectomycorrhizal species). Divergent distributions of CAZyme gene sets in various mycorrhizal lifestyles with an increasing repertoire of CAZyme from ectomycorrhizal *Scleroderma* species (in red) to saprotrophic brown rotters (higher; from left to right). Principal component analysis (PCA) was performed using the MycoCosm PCA tool (mycocosm.jgi.doe.gov). The CAZyme gene repertoires were obtained after semi-manual curation of protein sequences by the CAZy team (www.cazy.org)^17^.
